# Frank haematuria secondary to ruptured pseudoaneurysm of the inferior vesical artery: a case report and review of the literature

**DOI:** 10.1186/s13256-021-02668-x

**Published:** 2021-03-13

**Authors:** Mohammed Suoub, Ghassan Talahmeh, Salah Abdelraouf, Fadi Sawaqed

**Affiliations:** 1 Department of Special Surgery, Faculty of Medicine, Mut’ah University, Karak, 61710 Jordan; 2 Gardens Hospital, Amman, Jordan; 3 Jordan Hospital, Amman, Jordan

**Keywords:** Pseudoaneurysm, Haematuria, Embolization

## Abstract

**Background:**

Pseudoaneurysm of a vesical artery is an extremely rare iatrogenic complication; however, it may cause fatal haematuria.

**Case presentation:**

A 21-year-old Arab Jordanian male had multiple optical urethrotomies for an iatrogenic urethral stricture after he had rectal surgery for Hirschsprung’s disease at the age of 2 years. During one of his admissions to the Emergency Room (ER) with urinary retention, an attempt at suprapubic catheter insertion was complicated by massive bleeding at the insertion site of the catheter. Abdominal exploration showed a distended urinary bladder with clots and bleeding seen at the bladder neck that was controlled with multiple sutures. The patient rebled again two times; the first was controlled with cystoscopy and cautery for a pulsatile bleeder seen at the bladder neck. The second time, the patient required blood transfusion of three units of packed red blood cells. Angiography was performed, and a pseudoaneurysm at the base of the urinary bladder from the inferior vesical artery was diagnosed, which was controlled by embolization.

**Conclusion:**

Pulsatile bladder haemorrhage following urological intervention is suggestive of pseudoaneurysm or arteriovesical fistula, and angiography with embolization is recommended.

## Background

A pseudoaneurysm, also known as a false aneurysm, is leakage of arterial blood from an artery into the surrounding tissue with continuous communication between the originating artery and the resultant adjacent cavity.

Pseudoaneurysms may rupture externally to the skin or internally to an adjacent structure, such as the urinary bladder in our patient. Urgent diagnosis and management are required to prevent massive bleeding due to rupture of the pseudoaneurysm [1]. Massive haematuria with clots as a presenting symptom due to iatrogenic bladder pseudoaneurysm is an infrequent clinical entity, with few reported cases in the literature.

## Case presentation

A 21-year-old Arab Jordanian male patient had a history of Hirschsprung’s disease and surgery at the age of 2 years. During his surgery, he had an iatrogenic urethral injury initially treated with Foley catheter conservatively. Later, he started to have symptomatic recurrent urethral stricture because he underwent multiple optical urethrotomies.

In September 2019, the patient redeveloped symptoms of urethral stricture, and cysto-urethroscopy showed bulbomembranous urethral stricture, fibrosis in the external sphincter, and mild urinary bladder trabeculation.

Optical urethrotomy was performed, followed by catheter insertion for one week. Postoperative recovery was smooth, and the patient reported mild improvement in the urinary stream. Two months later, the patient presented to the Emergency Room (ER) with urinary retention. Suprapubic catheter (SPC) insertion was complicated by massive frank haemorrhaging at the site of catheter insertion with a large amount of pure blood seen in the urine bag. The patient was pale and anxious, and low blood pressure (100/50 mm Hg) and tachycardia (110 beats per minute) were recorded. The physical exam showed abdominal distention. He was immediately transferred to the operating room; under general anaesthesia, abdominal exploration was performed through a midline incision; the bladder was severely distended with blood clots, and after the evacuation of clots, an arterial bleeder was seen near the bladder neck over the trigon midway between the two ureteric orifices. After detailed visualization of both ureteric orifices, bleeding was controlled with multiple sutures followed by insertion of a suprapubic catheter and a Foley catheter for ten days.

**Fig. 1:**
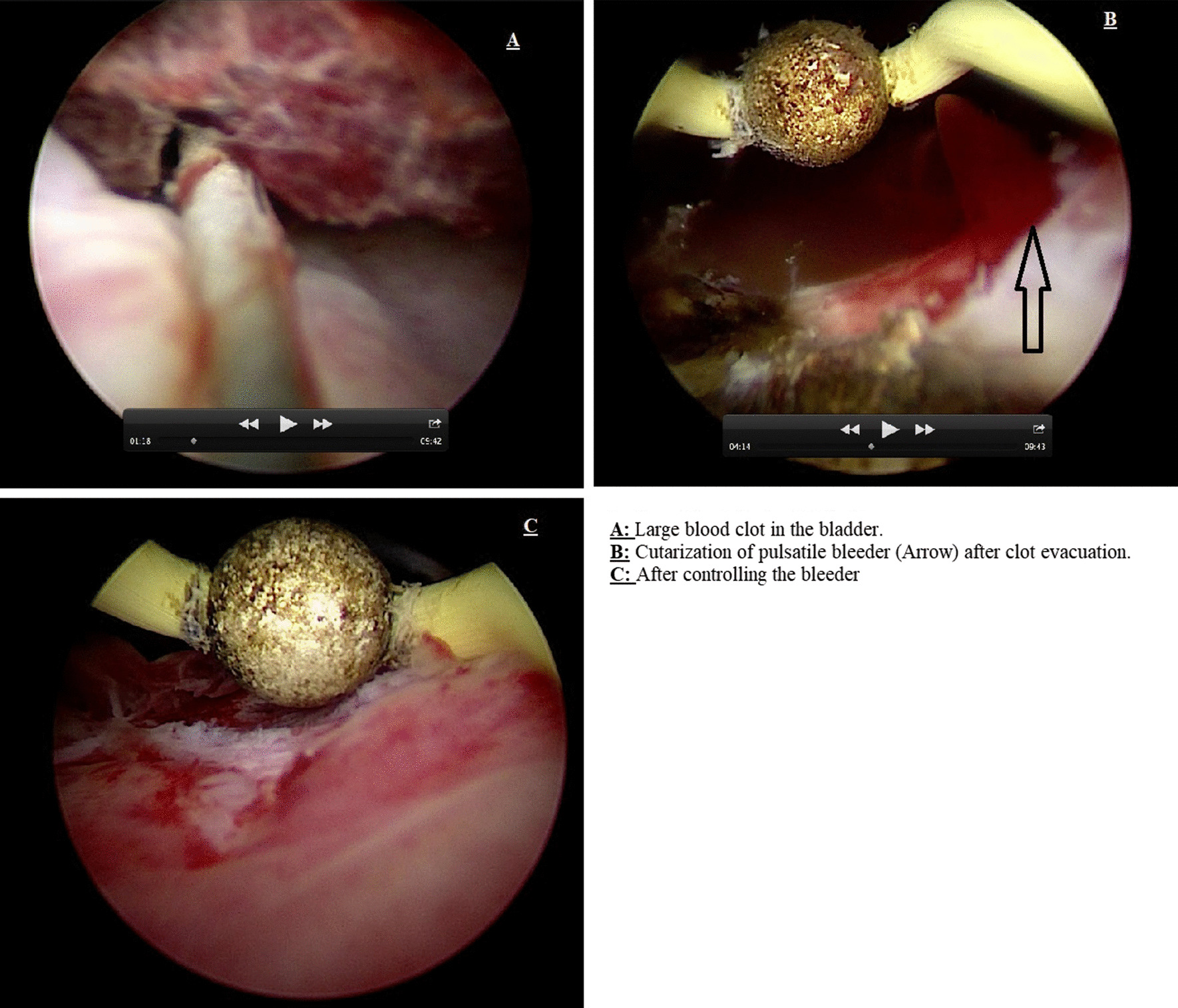
Second cysto-urethroscopy.

Eleven days after his discharge, the patient returned to the ER with gross haematuria. Cystoscopy revealed blood oozing at the bladder neck; electrocoagulation was successful in controlling his bleeding (Figure 1). One week later, he came to the ER with gross haematuria; a 3-way Foley catheter was inserted and kept on continuous irrigation with normal saline; he received three units of packed red blood cells; after two days, he failed conservative treatment, and bleeding persisted.

**Fig. 2:**
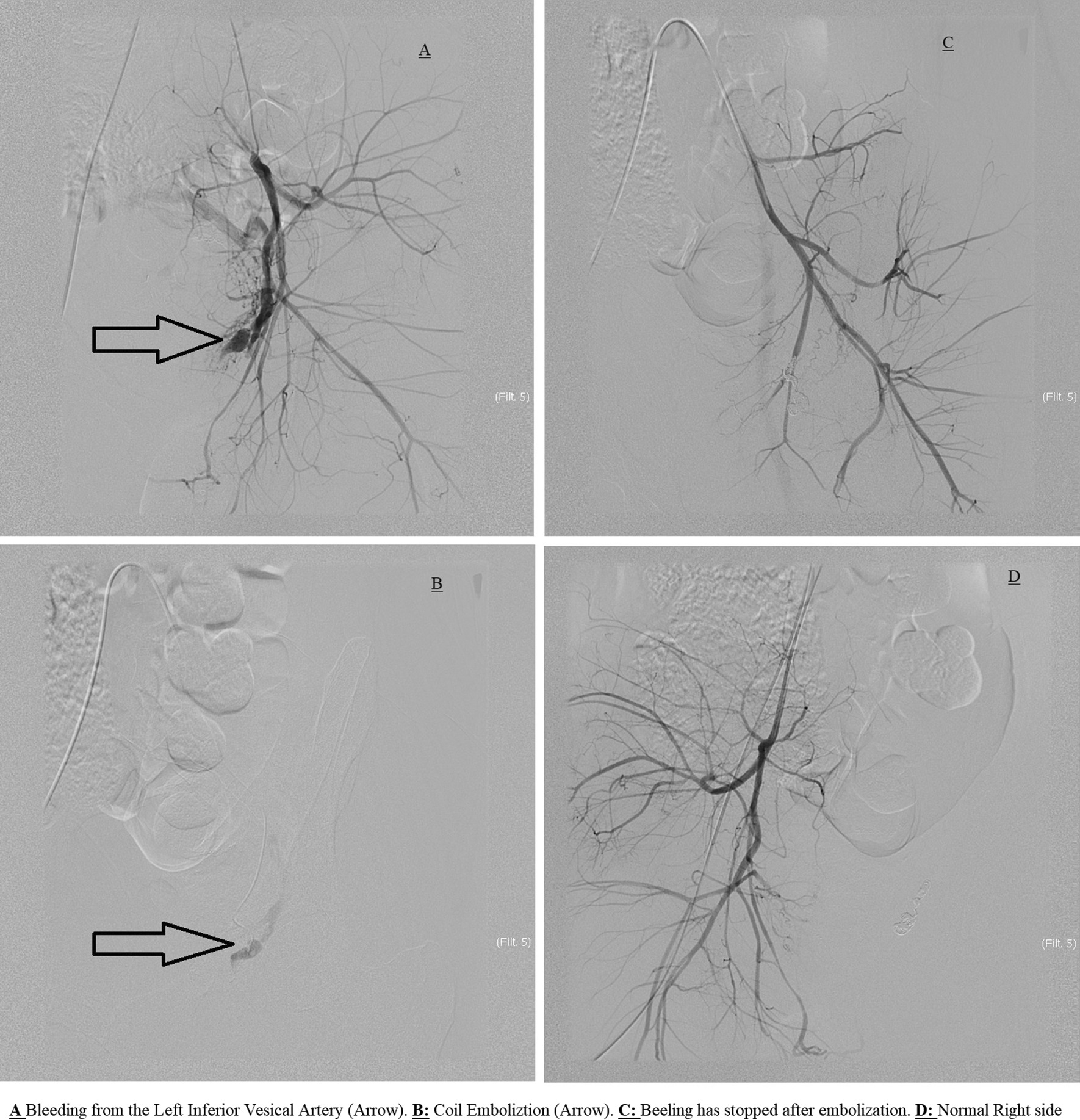
Angiography and embolization

Angiography was performed and identified bleeding from a pseudoaneurysm in the left inferior vesical artery at the base of the urinary bladder. Selective embolization of the feeding artery led to immediate control of bleeding (Fig. 2).

## Discussion

Our patient had traumatic suprapubic catheter insertion that penetrated through the posterior bladder wall, inducing massive bleeding and pseudoaneurysm formation secondary to injury to the inferior vesical artery at the base of the bladder.

Ichiya Honma reported that an elderly male patient with an internal iliac artery aneurysm had massive bleeding one week after he had cystoscopy from arteriovesical or arterio-ureteral fistula secondary to rupture of the internal iliac artery aneurysm [2].

Common causes of pseudoaneurysm of the urinary tract are iatrogenic injury, trauma [3], infection, or spontaneous. In 1978, Rous *et al*. reported the first case of a ruptured posttraumatic pseudoaneurysm of the external iliac artery one week after a gunshot wound to the lateral aspect of the bladder caused massive gross haematuria [4]. In a rare case, the rupture of a femoral pseudoaneurysm into the urinary bladder was observed in an IV drug abuser [5].

Levi reported massive haematuria secondary to a rupture of pseudoaneurysm in a patient after six months of aorto-bi-iliac artery bypass, which was complicated by a ureteral injury during the procedure [6].

Using cautery as first-line management in patients with frank haematuria and pulsatile bleeding from the urinary bladder is dangerous. Cautery may induce uncontrolled bleeding, or if bleeding subsides, the pseudoaneurysm will mostly likely bleed again, as seen in our patient. We recommend selective arterial embolization of the pseudoaneurysm, which controls bleeding immediately. Haegeman *et al*. reported pseudoaneurysms in 3 patients who presented with haematuria as a fistula between the iliac artery and neobladder pouch, and those patients were successfully managed with an arterial stent to exclude pseudoaneurysms [7].

Our patient had recurrent urethral stricture with multiple cysto-urethroscopy and optical urethrotomy. The multiple procedures and the primary disease of our patient may affect the urinary bladder wall, making it vulnerable to trauma and rupture.

To have safe SPC insertion and to avoid complications such as bowel injury and deep insertion of the SPC trocar into the posterior bladder wall, we advise urologists to use ultrasound-guided cystostomy tract creation and dilatation before SPC insertion. Perman Jacob *et al*. published a paper describing the use of ultrasound for SPC insertion and discussing the implications of its use [8].

## Conclusion

The development of a pseudoaneurysm of a vesical artery is extremely rare. This unique case presented as pseudoaneurysm that was not recognized at first and could have led to fatal haematuria after transurethral electrocoagulation for vesical bleeding. Further selective angiography revealed a pseudoaneurysm of the left inferior vesical artery with arteriovesical fistula formation. We postulate that a pulsatile bladder haemorrhage suggesting pseudoaneurysm or arteriovesical fistula should not be controlled with cauterization. Angiography with embolization is recommended in such cases.

## Data Availability

Not applicable
